# The Evolutionary History and Spatiotemporal Dynamics of the Fever, Thrombocytopenia and Leukocytopenia Syndrome Virus (FTLSV) in China

**DOI:** 10.1371/journal.pntd.0003237

**Published:** 2014-10-16

**Authors:** Xueyong Huang, Licheng Liu, Yanhua Du, Weili Wu, Haifeng Wang, Jia Su, Xiaoyan Tang, Qi Liu, Yinhui Yang, Yongqiang Jiang, Weijun Chen, Bianli Xu

**Affiliations:** 1 Center for disease control and prevention of Henan Province, Zhengzhou, China; 2 State Key Laboratory of Pathogens and Biosecurity, Institute of Microbiology and Epidemiology, Academy of Military Medical Sciences, Beijing, China; 3 Key Laboratory of Genome Sciences and Information, Beijing Institute of Genomics, Chinese Academy of Sciences, Beijing, China; University of Texas Medical Branch, United States of America

## Abstract

**Background:**

In 2007, a novel bunyavirus was found in Henan Province, China and named fever, thrombocytopenia and leukocytopenia syndrome virus (FTLSV); since then, FTLSV has been found in ticks and animals in many Chinese provinces. Human-to-human transmission has been documented, indicating that FTLSV should be considered a potential public health threat. Determining the historical spread of FTLSV could help curtail its spread and prevent future movement of this virus.

**Method/Principal Findings:**

To examine the pattern of FTLSV evolution and the origin of outbreak strains, as well to examine the rate of evolution, the genome of 12 FTLSV strains were sequenced and a phylogenetic and Bayesian phylogeographic analysis of all available FTLSV sequences in China were performed. Analysis based on the FTLSV L segment suggests that the virus likely originated somewhere in Huaiyangshan circa 1790 (95% highest probability density interval: 1756–1817) and began spreading around 1806 (95% highest probability density interval: 1773–1834). Analysis also indicates that when FTLSV arrived in Jiangsu province from Huaiyangshan, Jiangsu Province became another source for the spread of the disease. Bayesian factor test analysis identified three major transmission routes: Huaiyangshan to Jiangsu, Jiangsu to Liaoning, and Jiangsu to Shandong. The speed of FTLSV movement has increased in recent decades, likely facilitated by modern human activity and ecosystem changes. In addition, evidence of RNA segment reassortment was found in FTLSV; purifying selection appears to have been the dominant force in the evolution of this virus.

**Conclusion:**

Results presented in the manuscript suggest that the Huaiyangshan area is likely be the origin of FTLSV in China and identified probable viral migration routes. These results provide new insights into the origin and spread of FTLSV in China, and provide a foundation for future virological surveillance and control.

## Author Summary

FTLSV is novel bunyavirus belonging to genus Phlebovirus and was first found in Huaiyangshan area. The epidemiology and pathogenesis of FTLSV remain poorly understood. This lack of information underscores the importance of analyzing the movement and genetic history of FTLSV in China. Recent advances in Bayesian coalescent phylogenetic analyses have resulted in more sophisticated methods being available for determining the relative age of emerging pathogens. In this study, a phylogenetic and Bayesian phylogeographic analysis was performed for FTLSV whole genomic sequences isolated from China. Results identified the Huaiyangshan area as the most probable origin of FTLSV and suggested migrating routes of the virus. These results offer the first description of the movement and history of FTLSV in China. RNA segment reassortment was found in FTLSV; purifying selection appears to have been the dominant force driving the evolution of this virus. The results of this study could be used to facilitate the development of new strategies for controlling FTLSV.

## Introduction

In 2007, the fever, thrombocytopenia and leukopenia syndrome (FTLS; also called severe fever with thrombocytopenia syndrome, SFTS) was first reported in Henan province, China [1], [2]. Since then, this life-threatening disease has been reported in many Chinese provinces, including Hubei, Anhui, Jiangsu, Liaoning and Shandong. The main clinical symptoms are sudden onset of fever (>37.5°C), fatigue, marked anorexia, headache, myalgia, arthralgia, dizziness, lymphadenopathy, vomiting, and diarrhea. Laboratory tests show thrombocytopenia, leukopenia, increased urine protein, and elevated serum aspartate aminotransferase and alanine aminotransferase. Initially, this disease was diagnosed as suspended human granulocytic anaplasmosis (HGA) infection [3]. However, only 8% of patients were positive for HGA infection, indicating that other pathogens likely contribute to this syndrome. Samples collected in 2009 identified a novel virus in the *Bunyaviridae* family, genus *Phlebovirus* in FTLS cases; the virus has been named several times including severe fever with thrombocytopenia syndrome virus (SFSTV), Henan fever virus (HFV), fever, thrombocytopenia and leukopenia syndrome virus (FTLSV), and Huaiyangshan virus (HYSV) [1], [2], [4]. Serosurveillance has shown that 1 to3.8% of examined populations in hilly areas had FTLSV antibodies, suggesting that SFTSV has circulated widely in China but only a small proportion of infected individuals develop disease. The overall mortality of FTLSV infection in China is about 7.3% (2391 cases and 174 deaths),but ranges from 6.3% to 30% in various studies [5].

The genome of FTLSV is similar to that of other *Bunyaviridae*; it is composed of three segments, designated L, M and S. The L segment contains 6391 nucleotides encoding an RNA-dependent RNA polymerase; the M segment contains 3366 nucleotides encoding glycoproteins Gn and Gc; and the S segment contains 1760 nucleotides of antisense RNA encoding a nonstructural protein (NS) and a nucleocapsid protein (N) in opposite orientations. Partial sequencing of L and S segments from human cases and ticks confirmed that FTLSV is a novel *Phlebovirus* and is most closely related to the non-pathogenic Uukuniemi virus (the pairwise nucleotide similarities for the L, M and S segments are 34%, 24% and 29%, respectively) [4]. All FTLSV sequences currently available in GenBank are similar regardless of their sampling locations (>90% sequence similarity), suggesting that they descended from a relatively recent common ancestor.

Only a few members of the Phlebovirus genus use ticks as a vector; FTLSV is likely to be transmitted to human populations from an animal reservoir via feeding of infected ticks [6]. FTLSV has been detected in *Haemaphysalis longicornis* and *Rhipicephalus microplus* ticks collected from a number of domestic animals, including cattle, buffalo, goats, cats and dogs [4], [7], [8]. The potential for human-to-human transmission has also been shown [9], [10], [11], and phylogenetic analysis of partial L, M and S sequences has indicated that there are five phylogenetic groups circulating in China [4]. However, more detailed genetic information is needed to fully characterize the history of FTLSV and elucidate its transmission dynamics in China. Such studies could facilitate the future development of new strategies for controlling FTLSV.

In the present study, a powerful Bayesian phylogeographic approach to reconstruct the transmission of FTLSV through space and time was used to infer the geographic locations of ancestral viral lineages. In addition estimates of viral evolution rate and the divergence date of FTLSV were calculated.

## Materials and Methods

### Isolation and sequencing of viruses

Serum samples from FTLSV-infected patients (n = 12) were obtained by disease control and prevention workers in the Xinyang and Nanyang counties of Henan Province, China, during 2011–2012, and viral isolates were cultured from the sera [2]. All laboratory procedures involving viral cultures were performed under Biosafety Level-2 containment. All patients provided written informed consent for the research use of their samples. This research was approved by the Review Board of the Institute of Microbiology and Epidemiology [Beijing] and the Center for Disease Control and Prevention of Henan Province.

For sequencing, total RNA was extracted from 140 µl aliquots of cell culture supernatant obtained from each sample using a QIAamp viral RNA Mini Kit (Qiagen) according to the manufacturer's instructions. cDNA was reverse transcribed from 6 µl of RNA at 45°C for 50 min in a 20 µl solution containing 50 mM Tris–HCl (pH 8.3), 75 mM KCl, 3 mM MgCl_2_, 10 mM DTT, 100 ng of random hexamer primers, 200 units of Superscript II (Invitrogen), 25 units of RNasin (Promega) and 0.5 mM dNTPs. Overlapping PCR was performed, and products were subjected to Sanger sequencing [12]. Whole genome sequences were assembled using the SeqMan package in the DNASTAR 6.0 software.

### Sequence dataset

Sequences of FTLSV isolates available in the GenBank were downloaded and used in phylogenetic analyses. Only full-length S, M and L segment sequences with available collection dates and locations were included. The viral sequences that were newly determined in this study have been deposited in GenBank under accession numbers KF356517 to KF356552. Sequences were aligned by Clustal W using BioEdit software [13]. Recombination events were screened using the Recombination Detection Program (RDP) version 4.16 [14], under both the default and triplet settings. RDP implements a combination of methods, including RDP [14], CHIMAERA [15], BootSCAN [16], MAXCHI [17],GENECONV [18] and 3Seq [19] for recombination detection. Recombinant sequences were excluded before phylogenetic and phylogeographic analyses. A total of 84 S-segment sequences, 72 M-segment sequences, and 68 L-segment sequences were included in our analysis.

### Phylogenetic analysis

Phylogenetic trees for the three viral RNA segments were constructed using the maximum likelihood (ML) method, a feature of the software MEGA 5 [20], and the Bayesian MCMC method applied using MrBayes 3.2.1 [21]. The general time reversible (GTR) model with gamma distribution (GTR+G) was used for both methods. The reliability of the ML analysis was evaluated by a bootstrap test with 1000 replicates. The Bayesian MCMC method was run for 500,000 generations per segment.

### Analysis of evolutionary rates and divergence times

Evolution rates and estimates of time since the most recent common ancestor (MRCA) were generated via Bayesian coalescent phylogenetic analysis using the BEAST software package version 2.0.2 [22] and Tracer version 1.4 [23]. The jModelTest version 2.1.7 program [24] was used to examine possible nucleotide substitution models, and the most appropriate model for each dataset was selected based on Akaike's Information Criterion (AIC). GTR+G was selected for all three segments. Preliminary analyses were conducted to determine which clock (strict versus relaxed) and demographic (constant versus Bayesian Skyline population size) models were most appropriate for the datasets, with runs initially consisting of 10,000,000 generations. An analysis of marginal likelihoods [25] indicated that the relaxed uncorrelated lognormal clock [26] and constant population size model were most appropriate for all three segments (log10 Bayes Factors >2 in all cases). Thus, the final Bayesian coalescent analyses used the GTR+G substitution model, a relaxed uncorrelated lognormal clock, and a constant population size. The S and L segment datasets were run for 50,000,000 generations, whereas the M segment dataset was run for 40,000,000 generations to ensure effective sample sizes (ESSs) of at least 200. Maximum clade credibility trees were summarized using TreeAnnotator [23] and depicted using FigTree [23]. FTLSV (SFTSV) sequences from Japan (AB817979–AB818002) were excluded from this analysis because the isolation time was not provided.

### Phylogeographic analysis based on the L segment

To investigate the phylogeographic diffusion process along the posterior sets of the trees, relationships between locations were identified using the Bayesian stochastic variable search selection (BSSVS) applications in BEAST version 2.0.2 [22]. A Hasegawa-Kishino-Yano (HKY) substitution model, a constant population size tree prior, and a strict molecular clock were used. A standard discrete phylogeographic analysis using continuous-time Markov chains (CTMCs) to estimate the migration events between major geographic regions was set up [27]. This method can be used to infer the location state of the ancestral-branch geographic states, detect non-zero rates of state-transition over the whole tree, and build a reversible diffusion rate matrix between previously defined locations identified using the BSSVS applications. The results were summarized in an MCC tree, and further visualized by converting the tree into a keyhole markup language (KML) file suitable for viewing in Google Earth (http://earth.google.com). Following our discrete phylogeographic visualizations, we used SPREAD 1.04 [28] to estimate the putative spatiotemporal pattern of the spread and visualize the diffusion of the virus. The Bayes factor (BF) test, which determines statistically significant phylogeographic links, was performed using SPREAD 1.04. If BF>3, the phylogeographic link between two locations was considered to be statistically significant.

For phylogeographic analysis, each FTLSV sequence (or isolate/strain) was first assigned a character state reflecting its location of origin. Because the FTLS cases reported in Henan and Hubei province were located near the border of Tongboshan, belonging to the Huaiyangshan Mountain region, Henan and Hubei Provinces were combined into the Huaiyangshan (HYS) region in the analysis. Based on the geographic locations from which the studied isolates were obtained, migration was considered among four provinces (Anhui, Jiangsu, Shandong, and Liaoning) and one area (Huaiyangshan). For each taxon in the dataset, character states were assigned to the respective locations (summarized in Table 1). FTLSV strains isolated from Japan (AB817979–AB818002) were excluded from this analysis because isolation times were not available.

**Table 1:** Sequences of FTLSV with associated location of origin, collection dates, host and GenBank accession numbers.

Isolated strain	Year of isolation	Location	Host	NCBI Access number (L segment)	NCBI Access number (M segment)	NCBI Access number (S segment)
Phlebovirus_AH12_2010	2010	Anhui	Human	HQ116417	HQ141590	HQ141591
Phlebovirus_AH15_2010	2010	Anhui	Human	HQ141592	HQ141593	HQ141594
Phlebovirus_HB29_2010	2010	Hubei	Human	HM745930	HM745931	HM745932
Phlebovirus_HN13_2010	2010	Henan	Human	HQ141598	HQ141599	HQ141600
Phlebovirus_HN6_2010	2010	Henan	Human	HQ141595	HQ141596	HQ141597
Phlebovirus_JS2007_01	2007	Jiangsu	Human	JF837593	JF837594	JF837595
Phlebovirus_JS24_2010	2010	Jiangsu	Human	HQ830163	HM802201	HQ830165
Phlebovirus_JS6_2010	2010	Jiangsu	Human	HQ830169	HQ830170	HQ830171
Phlebovirus_JSD1_2011	2011	Jiangsu	Dog	JF267783	JF267784	JF267784
Phlebovirus_JS26_2010	2010	Jiangsu	Human	HQ830166	HQ830167	HQ830168
Phlebovirus_SD24_2010	2010	Shandong	Human	HM802200	HM802201	HM802205
Phlebovirus_SD4_2010	2010	Shandong	Human	HM802202	HM802203	HM802204
2010_FQM_2010	2010	Huaiyangshan	Human	HQ419227	HQ419236	HQ419240
2010_T112_2010	2010	Huaiyangshan	Human	HQ419228		
2010_WSQ_2010	2010	Huaiyangshan	Human	HQ419226		
2012_1_L_2012	2012	Henan	Human	KF356550	KF356539	KF356527
2012_2_L_2012	2012	Henan	Human	KF356551	KF356540	KF356528
AHL_China_2011	2011	Anhui	Human	JQ670934	JQ670930	JQ670932
AHZ_China_2011	2011	Anhui	Human	JQ670929	JQ670931	JQ670933
HB154_China_2011	2011	Hubei	Human	JQ733561	JQ733560	JQ733562
HB155_China_2011	2011	Hubei	Human	JQ733564	JQ733563	JQ733565
HB156_China_2011	2011	Hubei	Human	JQ733567	JQ733566	JQ733568
Henan_CHN_01_2010	2010	Henan	Human	HQ642766	HQ642767	HQ642768
Henan_CHN_20_2010	2010	Henan	Human	JF682773	JF682774	JF682775
Henan_CHN_69_2010	2010	Henan	Human	JF682776	JF682777	JF682778
JS2010_015_2010	2010	Jiangsu	Human	JQ317172	JQ317173	JQ317174
JS2010_018_2010	2010	Jiangsu	Human	JQ317175	JQ317176	JQ317177
JS2010_019_2010	2010	Jiangsu	Human	JQ317178	JQ317179	JQ317180
JS2011_004	2011	Jiangsu	Human	KC505123	KC505124	KC505125
JS2011_013_1	2011	Jiangsu	Human	KC505126	KC505127	KC505128
JS2011_027	2011	Jiangsu	Human	KC505129	KC505130	KC505131
JS2011_034	2011	Jiangsu	Human	KC505132	KC505133	KC505134
JS2011_062	2011	Jiangsu	Human	KC505135	KC505136	KC505137
JS2011_109	2011	Jiangsu	Human	KC505138	KC505139	KC505140
JS2012_020	2012	Jiangsu	Human	KC505141	KC505142	KC505143
JS2012_035	2012	Jiangsu	Human	KC505144	KC505145	KC505146
JS2012_goat01	2012	Jiangsu	Goat	KC473537	KC473538	KC473539
JS2012_tick01	2012	Jiangsu	Haemaphysalis longicornis	KC473540	KC473541	KC473542
L_HGX_2010	2010	Huaiyangshan	Human	HQ171187		
L_HZM_2010	2010	Huaiyangshan	Human	HQ171188		
L_WJ_2009	2009	Huaiyangshan	Human	HQ171186		
L_WJQ_2010	2010	Huaiyangshan	Human	HQ171190		
L_WWG_2010	2010	Huaiyangshan	Human	HQ171189		
LN3_2010	2010	Liaoning	Human	HQ141610		
Phlebovirus_JS3_2010	2010	Jiangsu	Human	HQ141601	HQ141602	HQ141603
Phlebovirus_JS4_2010	2010	Jiangsu	Human	HQ141604	HQ141605	HQ141606
Phlebovirus_LN2_2010	2010	Liaoning	Human	HQ141607	HQ141608	HQ141609
JS2010_014_2010	2010	Jiangsu	Human	JQ317169	JQ317170	JQ317171
SDLZtick12_2010	2010	Shandong	Haemaphysalis longicornis	JQ684871	JQ684872	JQ684873
SPL003A		Japan	Human	AB817980	AB817988	AB817996
SPL004A		Japan	Human	AB817981	AB817989	AB817997
SPL005A		Japan	Human	AB817982	AB817990	AB817998
SPL010A		Japan	Human	AB817983	AB817991	AB817999
SPL030A		Japan	Human	AB817984	AB817992	AB818000
SPL032A		Japan	Human	AB817985	AB817993	AB818001
SPL035A		Japan	Human	AB817986	AB817994	AB818002
XCQ-A112L	2010	Hubei	Haemaphysalis longicornis	JF906056	JF906057	
YG1		Japan	Human	AB817979	AB817987	AB817995
YGS1_2011	2011	Henan	Human	KF356549	KF356537	KF356525
YNY1_2011	2011	Henan	Human	KF356552	KF356538	KF356526
YPQ133_2011	2011	Henan	Human	KF356542	KF356530	KF356518
YPQ2_2011	2011	Henan	Human	KF356543	KF356531	KF356519
YPQ5_2011	2011	Henan	Human	KF356544	KF356532	KF356520
YSC19_2011	2011	Henan	Human	KF356548	KF356536	KF356524
YSC3_2011	2011	Henan	Human	KF356547	KF356535	KF356523
YSH39_2011	2011	Henan	Human	KF356541	KF356529	KF356517
YXX1_2011	2011	Henan	Human	KF356545	KF356533	KF356521
YXX2_2011	2011	Henan	Human	KF356546	KF356534	KF356522
2010_CBX	2010	Huaiyangshan			HQ419234	HQ419244
2010_FQM	2010	Huaiyangshan			HQ419236	
2010_LZR	2010	Huaiyangshan			HQ419237	HQ419243
2010_T112	2010	Huaiyangshan			HQ419238	
2010_WJ	2010	Huaiyangshan			HQ419229	
2010_WJQ	2010	Huaiyangshan			HQ4192301	
2010_WSQ	2010	Huaiyangshan			HQ419235	HQ419239
2010_WWG	2010	Huaiyangshan			HQ419231	
2010_WWX	2010	Huaiyangshan			HQ419232	HQ419242
2010_ZGQ	2010	Huaiyangshan			HQ419233	HQ419241
M_HZM_2010	2010	Huaiyangshan	Human		JF951393	
M_WSQ_2010	2010	Huaiyangshan	Human		JF951394	
Phlebovirus_LN3_2010	2010	Liaoning	Human		HQ141611	HQ141612
S_HGX_2010	2010	Huaiyangshan	Human			HQ171192
S_HZM_2010	2010	Huaiyangshan	Human			HQ171193
S_WJ_2009	2009	Huaiyangshan	Human			HQ171191
S_WJQ_2010	2010	Huaiyangshan	Human			HQ171195
S_WWG_2010	2010	Huaiyangshan	Human			HQ171194
SDLZCattle01_2011	2011	Shandong	Cattle			JQ693001
SDLZDog01_2011	2011	Shandong	Dog			JQ693003
SDLZP01_2011	2011	Shandong	Human			JQ693004
SDLZP02_2011	2011	Shandong	Human			JQ693005
SDLZP03_2011	2011	Shandong	Human			JQ693006
SDLZP04_201	2011	Shandong	Human			JQ693007
SDLZP05_2011	2011	Shandong	Human			JQ693008
SDLZP06_2011	2011	Shandong	Human			JQ693009
SDLZP07_2011	2011	Shandong	Human			JQ693010
SDLZP08_2011	2011	Shandong	Human			JQ693011
SDLZP09_2011	2011	Shandong	Human			JQ693012
SDLZSheep01_2011	2011	Shandong	Sheep			JQ693002
SDPLP01_2011	2011	Shandong	Human			JQ693013

### Phylogeny-trait association analysis

To determine the extent of geographic structure of FTLSV in China (by sampling locality), the association index (AI) and parsimony score (PS) statistics of clustering strength was computed using the BaTS (Bayesian tip-association significance testing) method [29] that examines all the plausible trees produced based on 60 L segment sequences of FTLSV strains by BEAST.

### Selection pressure analyses

Selection analyses were performed on the full dataset using the Datamonkey server implementation of HyPhy [30]. Single Likelihood Ancestor Counting (SLAC), Fixed Effects Likelihood (FEL), and Random-Effects Likelihood (REL) algorithms were used to assess the presence of selected sites in the L, M and S alignments. For the SLAC, FEL and IFEL results, a p-value cutoff of 0.05 was used; results of REL were considered significant if BF>100.0.

## Results

### Phylogenetic relationships of FTLSV genotypes

In this study, whole genome sequencing was performed on 12 FLTSV strains isolated in Henan Province, China. And the sequences were submitted into the Genebank (KF356517 to KF356552). Little variation was observed in the overall nucleotide lengths of the S, M, and L segments from viruses circulating in Henan from 2011 to 2012, and pairwise differences in the percent nucleotide identities were low, ranging from 0.1%–4.2%, 0.2%–4.7% and 0.1%–5.8% for the L, M, and S segments, respectively.

**Figure 1:**
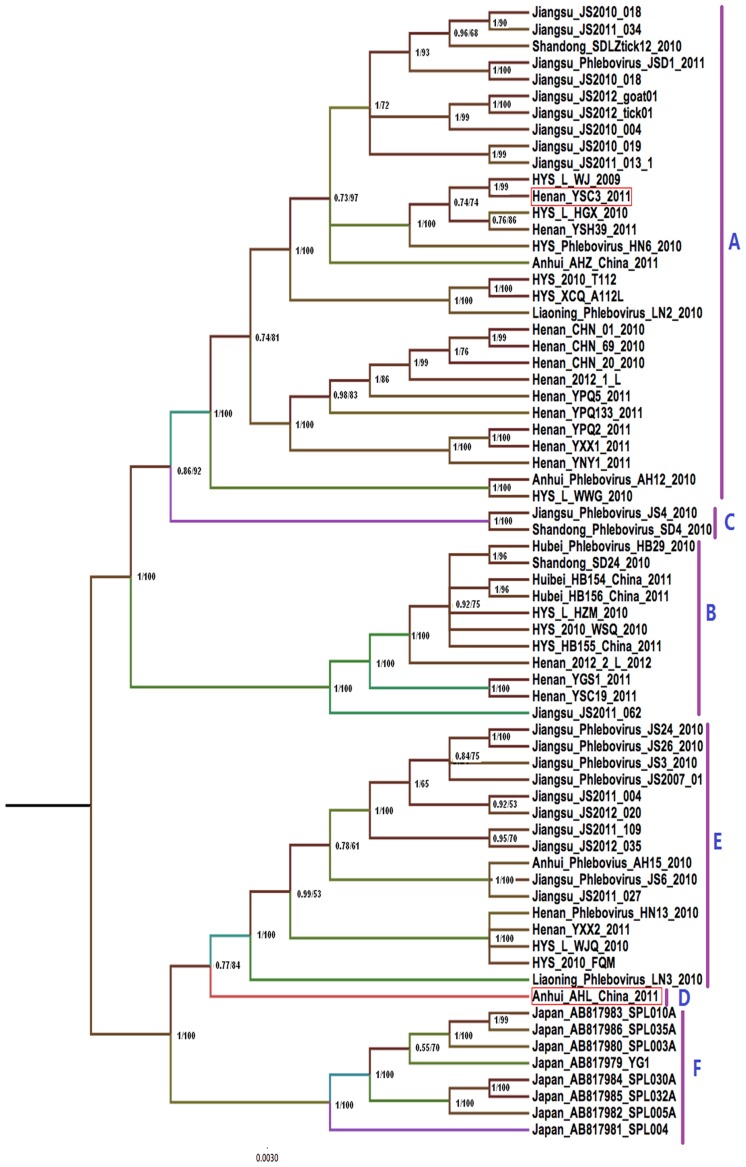
Bayesian coalescent and Maximum likelihood (ML) analysis of FTLSV based on the L segment. Maximum clade credibility and Maximum likelihood tree is shown with posterior probability values and bootstrap value depicted at the nodes. Phylogenetic analysis was carried out using Mrbayes 3.2.1 and Mega software 5, respectively. Designations of the different FTLSV phylogroups (A B,C,D,E,F clade) are as indicated, based on previously defined assignments. These sequences are described in more detail in Table 1. The sequences from Japan were included in this analysis.

**Figure 2:**
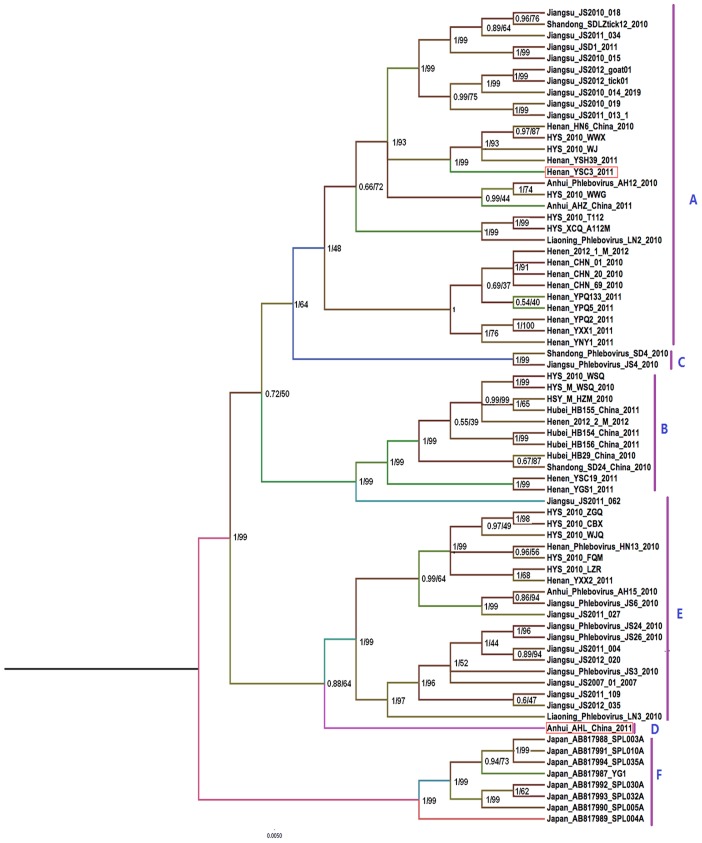
Bayesian coalescent and Maximum likelihood (ML) analysis of FTLSV based on the M segment. Maximum clade credibility and Maximum likelihood tree is shown with posterior probability values and bootstrap value depicted at the nodes. Phylogenetic analysis was carried out using Mrbayes 3.2.1 and Mega software 5, respectively. Designations of the different FTLSV phylogroups (A,B,C,D,E,F clade) are as indicated, based on previously defined assignments. These sequences are described in more detail in Table 1. The sequences from Japan were included in this analysis.

**Figure 3:**
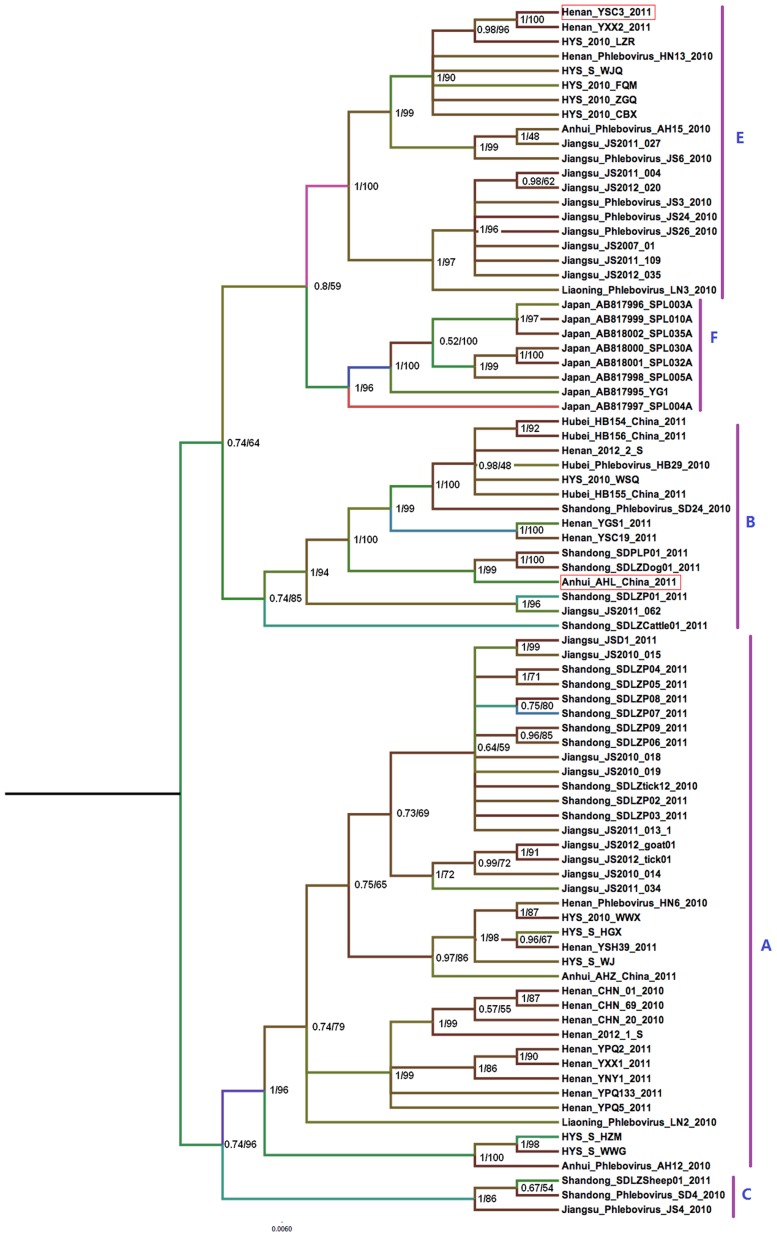
Bayesian coalescent analysis of FTLSV based on the S segment. Maximum clade credibility and Maximum likelihood tree is shown with posterior probability values and bootstrap value depicted at the nodes. Phylogenetic analysis was carried out using Mrbayes 3.2.1 and Mega software 5, respectively. Designations of the different FTLSV phylogroups (A,B,C,E,F clade) are as indicated, based on previously defined assignments. These sequences are described in more detail in Table 1. The sequences from Japan were included in this analysis.

For the three viral RNA segments, Bayesian analysis yielded highly supported tree topologies that were consistent with the ML results (Figures 1, 2 and 3). Phylogenetic analysis confirmed the presence of the five genotypes previously identified in China (designated A–E) [4], [31] and clustered the Japanese FTLSV strains, into a novel genotypic group. The Chinese genotypes were representive of the locations in China with the greatest burden of FTLS (Jiangsu, Henan, Liaoning, Shandong, Anhui, and Hubei Provinces and the Huaiyangshan region). Strains isolated from animals mainly clustered into the A lineage based on L and M analysis; however, the analysis of the S segments indicated that animal-isolated strains also included B and C genotypes. Strains isolated as part of this study were classified into A, B, D and E genotypes (Figures 1, 2 and 3).

### Evidence of RNA segment reassortment

Tree topologies obtained from the L segment analysis were similar to those generated using the M and S segments, but a more detailed analysis revealed some inconsistencies that seemed to indicate reassortment events. Two typical reassortment events appear to have occurred: 1) in the AHL/2011 strains, the L and M segments belonged to the D genotype, while the S segment fell into the B linage (Figures 1, 2 and 3); and in the YSC3 strains isolated from Henan, the L and M segments belonged to the A lineage, while the S segment belonged to the E lineage.

### Selection pressure on FTLSV

Analysis with the SLAC algorithm showed that the global ratio of nonsynonymous to synonymous nucleotide substitutions (dN/dS) in the L segment was 0.0480, suggesting the presence of predominantly purifying selection. The SLAC algorithm further identified 72 sites in the L segment that appeared to be under negative selection, while the FEL, IFEL and REL analyses detected one site (positions D691E/G) that appeared to be under positive selection in this segment. For the M segment, the SLAC algorithm analysis showed that the dN/dS was 0.0909 and detected 76 sites under negative selection. With respect to sites that appeared to be under positive selection, the FEL, IFEL and REL algorithms agreed on two sites (positions D37G/A and T501A/S); FEL and REL indicated one site (V323I); the REL algorithm alone detected one site (position I11T/V); and the IFEL method alone identified one site (T960I/S). For N and NS (encoded by the S segment), the dN/dS ratios were 0.0367 and 0.0991, respectively, and the SLAC, FEL, IFEL and REL methods failed to identify any sites that appeared to be under positive selection.

### Evolutionary rates and time since the MRCA of FTLSV

The mean molecular evolutionary rate estimates and 95% highest posterior density (HPD) intervals based on the Bayesian coalescent analyses were S = 3.17t10^−4^ (1.58×10^−5^–6.46×10^−4^), M = 3.08×10^−4^ (5.56×10^−5^–6.67×10^−4^) and L = 1.95×10^−4^ (5.01×10^−5^–3.37×10^−4)^ nucleotide substitutions/site/year. The MRCA of the current viruses identified today as FTLSV is estimated to have existed 117 (95%HPD = 36–241) years before present based the M segment analysis, 159 (95% HPD = 54–321) years before present based on the L segment analysis, and 195 (95% HPD = 28–455) years before present based on the S segment analysis.

### FTLSV probably originated from Huaiyangshan in the 18^th^ century

**Figure 4:**
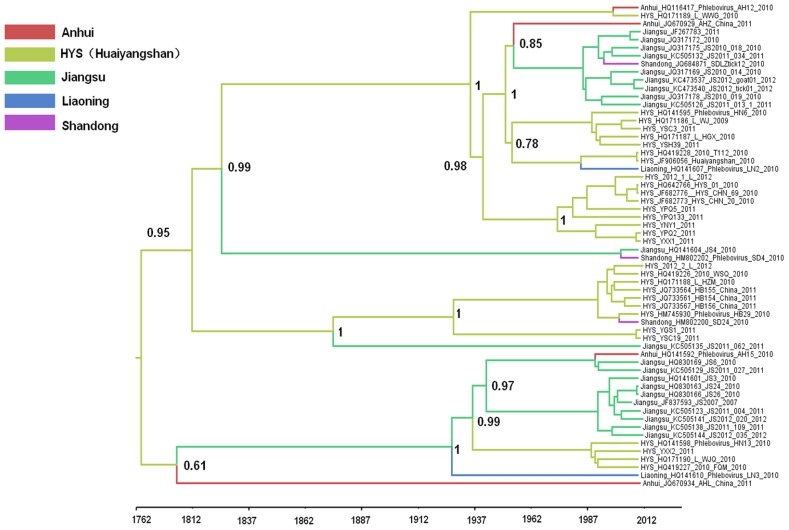
Maximum clade credibility tree based on L genes summarized for FTLSV were generated using the geospatial Bayesian analysis. Posterior clade probabilities for key nodes were shown. The colors of the branches correspond to their probable geographic location as calculated using the geospatial analysis.

**Figure 5:**
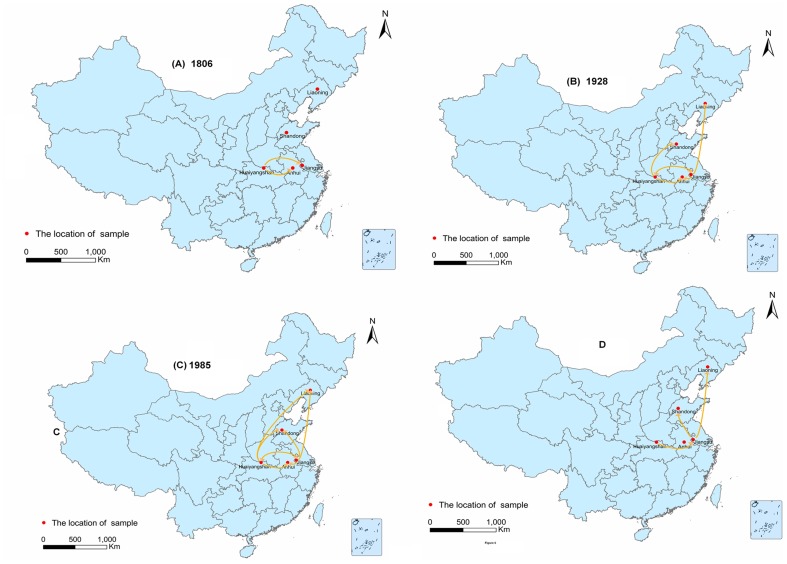
(A–C) Inferred migration graph for FTLSV in China and its reflection of the events reconstructed from the MCC tree for 1806, 1928 and 1985. The maps are based on satellite pictures made available in Google Earth. (D) The FTLSV migration routes inferred under the discrete phylogeographic diffusion model using a BF test. Rates supported by 3< = BF<10.

To determine the geographic origins and movements of FLTSV in China, the results of the Bayesian phylogeographic analyses were summarized by annotating the MCC tree nodes with their most probable location states via color labeling (Figure 4). The colors of the tree branches represent the most probable locations of their associated viral lineages (i.e., those supported by the highest probabilities), and a color change between two connected nodes implies a probable migration event. Simulative spatiotemporal pattern of FLTSV movements in China since the time of the MRCA was also visualized in Google Earth. The phylogeographic analysis showed the Huaiyangshan area appears to have been the origin of FLTSV in China (Figure 5). This was confirmed by the MCC tree, which showed that the MRCA of genotypes A–E originated in Huaiyangshan. The root state posterior probabilities for all locations ranged between 9.21% for Liaoning and 37.40% for Huaiyangshan. These dispersal histories are summarized in Figure 5, which shows snapshots of the dispersal patterns between locations from 1793 to 2012. Two main spread routes were observed (Figure 5A, Figure 5B, Figure 5C). FLTSV initially spread from the Huaiyangshan region into the surrounding provinces: eastward to Anhui and Jiangsu Provinces, and northward to Liaoning and Shandong Provinces. Thereafter, FTLSV is predicted to have spread from the Jiangsu Province to Liaoning, Shandong, and Anhui Provinces. In a further investigation of these diffusion patterns, the BF test identified three well-supported (BF>3) migration routes for FTLSV (Figure 5D): from Huaiyangshan to Jiangsu (BF = 9.74), from Jiangsu to Shandong (BF = 3.35), and from Jiangsu to Liaoning (BF = 3.17).

### The extent of geographic structure in the FTLSV L phylogeny

Both the AI and PS statistics indicated that there was very strong geographic clustering of FTLSV strains by province or area of origin (P<0.05) (Table 2). When the extent of phylogenetic clustering of individual province was tested, geographic clustering was significant for Huaiyangshan and Jiangsu except for Anhui, Liaoning, and Shandong, for which there was evidence of significant gene flow to and from other regions of China (P>0.05). These results suggest that the genetic diversity of FTLSV in Huaiyangshan and Jiangsu is shaped primarily by *in situ* evolution rather than extensive migration. However, the number of sequences from Anhui, Liaoning and Shandong was small, which may bias the result.

**Table 2:** Extent of geographic clustering, as indicated by AI and PS statistics.

	Observed value Null value			Null value			P value
Statistic	Mean	Lower 95% CI	Upper 95% CI	Mean	Lower 95% CI	Upper 95% CI	
Among province or Area							
AI	1.18	1.02	1.36	4.22	3.13	5.05	0.00
PS	13.00	13.00	13.00	24.98	22.36	27.83	0.00
Anhui vs others							
AI	0.48	0.33	0.59	0.82	0.41	1.21	0.21
PS	4.00	4.00	4.00	3.90	3.00	4.00	1.00
Huaiyangshan vs others							
AI	0.61	0.58	0.69	3.38	2.42	4.38	0.00
PS	7.01	7.00	7.00	20.41	17.60	23.81	0.00
Jiangsu vs others							
AI	0.58	0.43	0.68	3.16	2.36	3.97	0.00
PS	6.02	6.00	6.00	17.74	15.44	20.00	0.00
Liaoning vs others							
AI	0.08	0.08	0.08	0.43	0.10	0.63	0.02
PS	2.00	2.00	2.00	2.00	2.00	2.00	1.00
Shandong vs others							
AI	0.62	0.60	0.70	0.63	0.25	0.91	0.43
PS	3.00	3.00	3.00	2.95	3.00	3.00	1.00

## Discussion

FTLS represents a threat to public health, and understanding the how the causative virus sustains and spreads in the environment is important to implementing effective prevention and control measures. Currently, however, the mechanisms underlying the emergence and spread of FTLSV are unknown. The virus was first reported in 2007 [9]; at least 11 provinces in China including Beijing [32], and three countries, Korea [33], Japan (AB817979–AB818002) FTLSV-infected cases These reports demonstrated the regional threat of this pathogen and indicate that enhanced surveillance is needed to gain a better understanding of its evolutionary history, geographic spread and evolving virulence. Using a powerful and robust Bayesian phylogenetic and phylogeographic approach, we reconstructed the spatial and temporal spread of FTLSV in China.

The phylogenetic relationships between strains circulating in China and Japan were reconfirmed as a part of this study, as were identification of the five genotypes already identified as circulating in China, consistent with the results reported by Zhang and Lam et al [4], [31]. For our comparison, FTLSV sequences were mainly derived from viruses isolated from Henan, Hubei, Jiangsu, Anhui, Liaoning and Shandong Provinces, and the close phylogenetic relationship among them indicated that the movement of FTLSV is common in China. At least two possible reasons for unencumbered movement of FTLSV: 1) there are no obvious physical barriers to prevent viral transmission, as the locations all correspond to plains or hilly regions of China; and 2) these regions share similar enzootic vectors and potential primate and non-primate reservoirs. Several papers have reported the isolation of FTLSV from two tick species (*Haemaphysalis longicornis* and *Rhipicephalus microplus*) and domestic animals such as sheep, goat and cattle [4], [7], [8]. Furthermore, the analysis performed in this study suggest that FTLSV strains from Japan share a common ancestor with viral genotypes circulating in China, indicating that the virus has crossed the sea between China and Japan, forming the Japanese linage.

Evolution of FTLSV has impacted the sequence of all three viral RNA segments, creating unique viral genotypes and S, M and L RNA segment phylogenetic topologies indicate that FTLSV reassortment (particularly in the S segment) is not uncommon. Genetic reassortment can profoundly affect the pathogenicity and diversity of RNA viruses [34], [35], [36], [37], [38], [39]. For example, reassortment among influenza virus strains (another RNA virus with a segmented genome) results in antigenic shift and the emergence of new pandemic strains [40]. The S segment of FTLSV encodes the N protein, which binds viral RNAs and encapsidates them in to ribonucleoprotein complexes (RNPs) as well as the NS protein, which prevents the host interferon response by interfering with the cellular mRNAs and protein synthesis machineries of host cells. Reassortment of the S segment, therefore, could influence the replication and immunogenicity of the virus. The existence of many circulating genotypes in China and the wide distribution of potential vectors may increase the opportunity for reassortment to occur in FTLSV. Surveillance for detection of viruses with enhanced pathogenicity is critical to implementing control and prevention measures reducing the impact of FTLSV infection.

Purifying selection also appears to be another potential force in the evolution of FTLSV, although some sites were also found to be under positive selection. These results indicated that nucleotide fixation events are due primarily to genetic drift and stochastic processes, which may be important in the evolution of FTLSV.

Strong purifying selection can maintain evidence of sequence homology long after saturation has occurred at synonymous sites [41], [42]. But purifying selection can obscure the age of viral lineages (e.g., as observed with measles, Ebola, and avian influenza viruses) [41], [42]. This issue is a potential limitation to the work reported here. Unfortunately, there is not a answer to the problem of underestimated the tMRCA due to purifying selection in molecular clock analysis of virus evolution. However, with advances currently being made in the development of increasingly more realistic evolutionary models, the bias imposed by the purifying selection may be solved in the foreseeable future. Still, given the apparent recent history of FTLSV, it is possible that the presence of purifying selection may not have a significant impact of the current evolution of the virus.

Although the emergence of FTLSV and its association with FTLS was first confirmed during the summer of 2009 [1], similar symptoms had been reported by hospitals near Huaiyangshan since 2007 [1]. Consistent with this, these results indicate that FTLSV has been circulating in China for many years. The reconstructed epidemic history of FTLSV suggests that the virus is a recently emerged pathogen that originated around the 18^th^ century. Bayesian analysis indicated that the most recent common ancestor of the FTLSV strains existed an estimated 117 to 195 year prior to 2012. Similar estimates were obtained for the L, M and S segments, indicating that these segments have co-evolved. Lam et al. [31] reported that FTLSV originated from a common ancestor and most likely existed between 1817 and 1895, which is somewhat more recent estimate than the one proposed in this analysis. This difference might be explained in two ways. First, the evolutionary rate of Rift Valley Fever Virus (RVFV) was used as a model for the FTLSV analysis of Lam et al. Although RVFV and FTLSV both belong to the family *Bunyaviridae*, RVFV is mosquito-borne while FTLSV is tick-borne, and mosquito-borne RNA viruses are believed to evolve more rapidly than tick-borne viruses within the same family [43], [44]. Second, our analysis used full-length sequences of the three segments, and included many more sequences than were available in GenBank at the time of the previous study.

Bayesian phylogeographic analysis indicated that the Huaiyangshan area might be the origin of FTLSV. Huaiyangshan Mountain borders \ Henan and Hubei Provinces in China; these provinces are the source of the Huaihe River, which forms the dividing line between southern and northern China. This biologically diverse region has been and remains a home to cultural and economic exchanges between the two halves of China. As FTLSV has been detected in the circulating tick vectors *Haemaphysalis longicornis* and *Rhipicephalus microplus*, which are widely distributed across China and globally, conditions exist for FTLSV to have been transmitted from Huaiyangshan to neighboring provinces in China [4]. BaTS analysis detected strong structuring of the FTLSV phylogeny by provinces or area, suggesting that *in situ* evolution has played an important role in the maintenance of FTLSV in China. Additionally, the results of the BaTS analysis that considered the extent of population subdivision between individual provinces or areas and all others as a group indicate that for two of the five provinces or areas sampled (i.e., Huaiyangshan, and Jiangsu), the observed FTLSV genetic diversity was shaped primarily by *in situ* evolution as opposed to extensive migration.

This paper offers the first description of the movement of FTLSV in China. Our results indicate that this movement was slow during the early years of the spread, but has increasingly become more rapid in recent decades. Bayesian phylogeographic analysis showed that FTLSV arose in Huaiyangshan between 1756 and 1817, and then moved into the Jiangsu and Anhui Provinces between 1773 and 1834. Over the next hundred years, FTLSV continued to circulate through Jiangsu, Anhui and the Huaiyangshan region. Once FTLSV reached Jiangsu Province, this province became a secondary source of FTLSV spread, as the virus moved from Jiangsu Province into Liaoning Province between 1905 and 1943. In the past 50 or 60 years FTLSV has moved more rapidly, as the MCC tree shows that many strains isolated from Shandong, Liaoning and Anhui were recently introduced from Huaiyangshan and Jiangsu. Finally, the Bayesian test provided statistical support for three transmission routes: from Huaiyangshan to Jiangsu, from Jiangsu to Shandong and from Jiangsu to Liaoning.

Collectively, these findings indicate that Huaiyangshan and Jiangsu may be the two major origins of FTLSV in China. Movement over large distances was observed, such as that between Liaoning and Jiangsu Province and Huaiyangshan (∼1500 km). It is likely that an animal infected with FTLSV or carrying virus-infected ticks introduce FTLSV into Liaoning Province. The recent dramatic increases in unregulated wildlife trade, livestock import/export, and global human movement are likely to have created additional opportunities for spread of this severe disease. In addition, expanding urban populations have triggered ecosystem changes that may have promoted the emergence of FTLSV by increasing human exposure to the natural reservoir or vector.

In conclusion, our results provide new insights into the origin and spread of FTLSV in China. Further studies are needed to obtain complete FTLSV genomes from other countries in which the virus is endemic, and to elucidate the dynamics of the worldwide dispersion of FTLSV.
